# Unmodified Silica Nanoparticles Enhance Mechanical Properties and Welding Ability of Epoxy Thermosets with Tunable Vitrimer Matrix

**DOI:** 10.3390/polym13183040

**Published:** 2021-09-09

**Authors:** Anna I. Barabanova, Egor S. Afanas’ev, Vyacheslav S. Molchanov, Andrey A. Askadskii, Olga E. Philippova

**Affiliations:** 1 A.N. Nesmeyanov Institute of Organoelement Compounds, Russian Academy of Sciences, 119991 Moscow, Russia; nambrot@yandex.ru (E.S.A.); andrey@ineos.ac.ru (A.A.A.); 2 Physics Department, Moscow State University, 119991 Moscow, Russia; molchan@polly.phys.msu.ru (V.S.M.); phil@polly.phys.msu.ru (O.E.P.); 3 Moscow State University of Civil Engineering, 129337 Moscow, Russia

**Keywords:** vitrimer, epoxy network, nanocomposite, silica nanoparticles

## Abstract

Epoxy/silica thermosets with tunable matrix (vitrimers) were prepared by thermal curing of diglycidyl ether of bisphenol A (DGEBA) in the presence of a hardener—4-methylhexahydrophthalic anhydride (MHHPA), a transesterification catalyst—zinc acetylacetonate (ZAA), and 10–15 nm spherical silica nanoparticles. The properties of the resulting material were studied by tensile testing, thermomechanical and dynamic mechanical analysis. It is shown that at room temperature the introduction of 5–10 wt% of silica nanoparticles in the vitrimer matrix strengthens the material leading to the increase of the elastic modulus by 44% and the tensile stress by 25%. Simultaneously, nanoparticles enhance the dimensional stability of the material since they reduce the coefficient of thermal expansion. At the same time, the transesterification catalyst provides the thermoset with the welding ability at heating, when the chain exchange reactions are accelerated. For the first time, it was shown that the silica nanoparticles strengthen welding joints in vitrimers, which is extremely important, since it allows to repeatedly use products made of thermosets and heal defects in them. Such materials hold great promise for use in durable protective coatings, adhesives, sealants and many other applications.

## 1. Introduction

Vitrimers represent a new class of polymer materials first proposed 10 years ago by L. Leibler and co-workers [1,2,3]. They consist of covalent adaptable networks that can rearrange their topology via exchange reactions while keeping the cross-link density unchanged. The first vitrimers were based on epoxy networks undergoing transesterification reactions [1,3,4]. Later, many other exchange reactions were proposed [2,5] including transamination of vinylogous urethanes [2,5] transalkylation of triazolium salts [6], olefin metathesis [7,8], thermo-activated disulfide rearrangements [9] and others.

Vitrimers, like other thermosets, possess excellent mechanical properties, enhanced chemical resistance and thermostability [10]. At the same time, the exchange reactions provide to vitrimers some additional features, such as the ability for reprocessing, recycling, reshaping and healing, which are quite important both from economic and ecological points of view. Moreover, exchange reactions enable the surface welding of vitrimers [1,11,12,13]. Welding being a viable joining technique gives the opportunity to produce multilayered structures combining different materials with desirable properties. Before the invention of vitrimers, the welding was restricted only to thermoplastics, which can be welded, when they are softened (e.g., by heating above their melting point). Vitrimers opened the way to weld thermosets due to exchange reactions that provided high enough interfacial diffusion of polymer chains [12] in order to form the welded joint.

Addition of nanoparticles is a promising way to improve the properties of vitrimers. One can expect that nanoparticles possessing a large surface area to interact with polymer chains are able not only to enhance the mechanical properties and the resistance to weathering [14,15] of vitrimers, but also to facilitate the welding through the intermolecular interactions. However, by now, there are only few papers describing vitrimer nanocomposites. They concern mainly vitrimers with epoxy matrix. This is not unexpected because among different vitrimers, epoxy thermosets are of particular interest [16], since epoxy resins are the most used thermoset materials [17], which share ~70% of the global thermosets market [4]. They possess many valuable properties such as high adhesive strength, good chemical resistance, and low curing shrinkage [16]. The epoxy vitrimer nanocomposites studied by now were filled with silica [18], graphene oxide [19], cellulose nanocrystals [20], carbon black [21], carbon fiber [22] or carbon nanotubes [11]. It was shown that nanoparticles can increase the elastic modulus [18,19] and enhance the self-healing and self-repairing ability of vitrimers [19].

Among different fillers, silica nanoparticles have many advantages including low cost, reach possibilities to modify their surface functionality, and easily tunable size. However, to the best of our knowledge, there is only one paper devoted to epoxy/silica vitrimer nanocomposites [18]. It concerns so-called soft nanocomposites with rather low *T*_g_. No studies of hard epoxy vitrimer nanocomposites with strongly cross-linked matrix are available by now. At the same time, such nanocomposites are very promising for various applications including durable protective coatings, adhesives, paints, sealants, windmill blades and so forth [16,23,24,25]. For instance, they are required in civil infrastructure applications as adhesives for joining composite materials or repairing civil structures [15].

Therefore, in this article, we studied hard silica nanocomposite thermosets made from an epoxy-based vitrimer. The vitrimer was prepared by curing one of the most extensively used commercial epoxy resins [26]—bisphenol A diglycidyl ether (DGEBA) with 4-methylhexahydrophthalic anhydride (MHHPA) in the presence of a transesterification catalyst zinc acetylacetonate (ZAA). The topology rearrangements in the vitrimer thus obtained result from thermoactivated and catalyzed transesterification reactions. We demonstrate that the mechanical properties of hard epoxy-based vitrimers can be reinforced with 10–15 nm spherical silica nanoparticles without preventing the topology rearrangements. Moreover, the nanofiller strengthens the welding joints, which opens wide perspectives for the development of welding of thermoset vitrimers allowing repairing, reprocessing or combining them with other materials.

## 2. Materials and Methods

### 2.1. Materials

**Figure 1:**
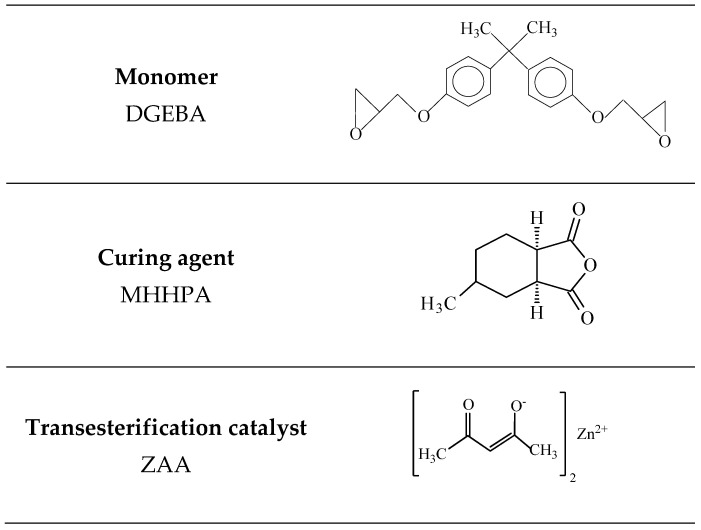
Chemical structure of the monomer bisphenol A diglycidyl ether DGEBA, curing agent 4-methylhexahydrophthalic anhydride MHHPA, and transesterification catalyst zinc acetylacetonate ZAA.

Monomer DGEBA, transesterification catalyst ZAA from Sigma-Aldrich (Steinheim am Albuch, Baden-Wurttemberg, Germany) and curing agent MHHPA (purity 98%) from Acrus Organics (Geel, Antwerp, Belgian) were used without further purification. Their chemical structures are presented in Figure 1. Silica nanoparticles in the form of 30–31 wt% colloidal dispersion in methyl ethyl ketone (MEK-ST) were purchased in Nissan Chemical Corporation (Santa Clara, CA, USA) and used as received. The size of silica particles provided by the supplier is 10–15 nm.

The content of surface silanol Si–OH groups was estimated by two complementary techniques: potentiometric titration and volumetric method. The first method is based on potentiometric titration of excess sodium hydroxide after its interaction with Si–OH groups on the surface of nanoparticles for 1 day with stirring [27]. The volumetric method is based on measurement of volume of gaseous products of reaction of silanol groups Si–OH with Grignard reagent (CH_3_MgI in diisoamyl ether) [28]. The number of silanol groups estimated by the volumetric method was equal to 0.45 mmol/g, which is close to the value obtained by titration (0.5 mmol/g).

### 2.2. Synthesis of Epoxy/Silica Thermosets with Vitrimer Matrix

Samples of epoxy/silica thermosets with vitrimer matrix were prepared according to the method developed in [29]. ZAA powder (0.2134 g) was added to DGEBA (2.7866 g), and the mixture was homogenized at 150 °C for 3 h under stirring. Then, the SiO_2_ colloid dispersion MEK-ST (0.9912 g) was added and the resulting mixture was heated at 80 °C for 2 h. After evaporation of the organic solvent MHHPA (2.6879 g) was added, the reaction mixture was stirred at room temperature and poured into a Teflon mold mounted on a horizontal plate in a Binder drying oven. The temperature was gradually increased (5–6 °C/min) to 140 °C and the reaction mixture was kept at this temperature for 12 h. The conditions of preparation of epoxy/silica networks with vitrimer matrices are given in Table 1.

The degree of curing α of the epoxy networks was determined using differential scanning calorimetry data obtained on a NETZCH DSC 204F1 instrument (Netzsch, Selb, Bayern, Germany) and calculated using the formula [30]:

(1) $$\alpha =\frac{\Delta H_{T}-\Delta H_{R}}{\Delta H_{T}}\times 100\%$$

where Δ*H*_T_ is the amount of heat released during the nonisothermal DSC scanning of the initial reaction mixture and Δ*H*_R_ is the residual amount of heat released during the DSC scanning of the cured sample.

### 2.3. Mechanical Properties

Tensile tests of the native samples were performed on Lloyd LS5, AMETEK STC (Lloyd Instruments Ltd., Segensworth, Hampshire, UK) tensile machine at room temperature. Stress–strain curves were obtained on rectangular samples (1.8 × 4 × 37 mm) with a 5 kN load cell at constant cross-head speed of 2 mm/min and effective gauge length of 16 mm. The elastic modulus *E* was determined from the slope of the initial linear section of elastic deformation ε which the material undergoes under loading: *E* = σ/ε.

### 2.4. Thermomechanical Analysis

The thermomechanical analysis (TMA) of epoxy thermosets was performed on a TMA Q400 instrument (TA Industries, Woodland, CA, USA). Thermomechanical curves were recorded at a constant load of 100 g in the penetration mode using the probe with a diameter of 2.54 mm during heating in air from room temperature to 350 °C at a constant speed of 5 °C/min. The *T*_g_ value was determined as the mid-point of the temperature range of the transition of the sample from the glassy to rubbery state. The coefficient of linear thermal expansion (CTE) was measured in the expansion mode from slopes of the TMA curve before and above *T*_g_. In the experiments, the samples were first heated to 250 °C and kept at this temperature for 10 min, then cooled to 25 °C, and again heated to 250 °C at a rate of 5 °C/min. The values of *T*_g_ and CTE were determined from the second heating cycle [31] to avoid the influence of the thermal and mechanical history of epoxy thermosets on their thermal properties.

The values of *T*_g_, storage *E*′, and loss *E*″ moduli of cured samples were determined with a dynamic mechanical analyzer NETZCH DMA 242 E Artemis (Netzsch, Selb, Bayern, Germany). The samples were cut to the size of 30 × 6 × 2 mm before being mounted on a single cantilever clamp and measured at a frequency of 1.0 Hz and a heating rate of 1 °C/min from 25 to 250 °C. The storage modulus, loss modulus and loss factor, tan δ, were calculated as a function of temperature over a range from 25 to 250 °C. The glass transition temperature *T*_g_ was determined as a temperature corresponding to the peak value of tan δ.

### 2.5. Fourier-Transform Infrared Spectroscopy (FTIR)

FTIR spectroscopy measurements were carried out on Bruker Vertex 70v FTIR spectrometer (Bruker Optic GmbH, Ettlingen, Baden-Wurttemberg, Germany) in attenuated total reflectance (ATR) mode using a PIKE GladyATR device with diamond ATR unit. The FTIR-ATR spectra were recorded in 4000–400 cm^−1^ range with a resolution of 4 cm^−1^. The samples were firmly pressed to the surface of the ATR unit by means of a special clamping device.

### 2.6. Investigation of the Ability of Epoxy Vitrimer Nanocomposite for Welding

The ability of epoxy vitrimer nanocomposites for welding was studied as follows. Rectangular samples (1.8 × 4 × 37 mm^3^) were superimposed on each other. To ensure a good contact between the samples, they were placed in a special clamping device, compressed by ~25%, and left in a drying oven at a temperature of 160 °C for 5–6 h. The overlap area of the rectangular samples was 5 × 5 mm = 25 mm^2^. Tensile testing of the welded joints of the samples was carried out on Lloyd LS5, AMETEK STC tensile machine at a speed of 5 mm/min.

## 3. Results and Discussion

### 3.1. Mechanical and Thermal Properties of Epoxy/Silica Vitrimer Nanocomposites

The epoxy/silica vitrimer nanocomposites were prepared by mixing 30 wt% colloid dispersion of 10–15 nm spherical silica nanoparticles in MEK with DGEBA and ZAA as the interchain exchange catalyst (Figure 1). The surface silanol groups of the nanoparticles can interact with the epoxy network through H-bonding. It should be noted that no curing catalyst was used during the synthesis. The mechanism of noncatalyzed reaction of aromatic epoxides such as DGEBA with anhydrides is well-studied in the literature [32,33,34,35]. It was shown that it starts by the interaction of OH-groups, which are present as impurity in epoxy oligomers, with anhydride giving monoester with a free carboxylic acid group, which, in turn, reacts with an epoxy group forming a diester and a new OH-group that can react with the anhydride (Figure S1, Supplementary Materials).

Conditions of preparation of the epoxy/silica networks are listed in Table 1. The nanocomposites obtained did not dissolve being immersed in trichlorobenzene at 160 °C for 16 h, which confirmed that the polymer is cross-linked, and the exchange reactions occurring in the presence of transesterification catalyst do not induce depolymerization of the network.

**Figure 2:**
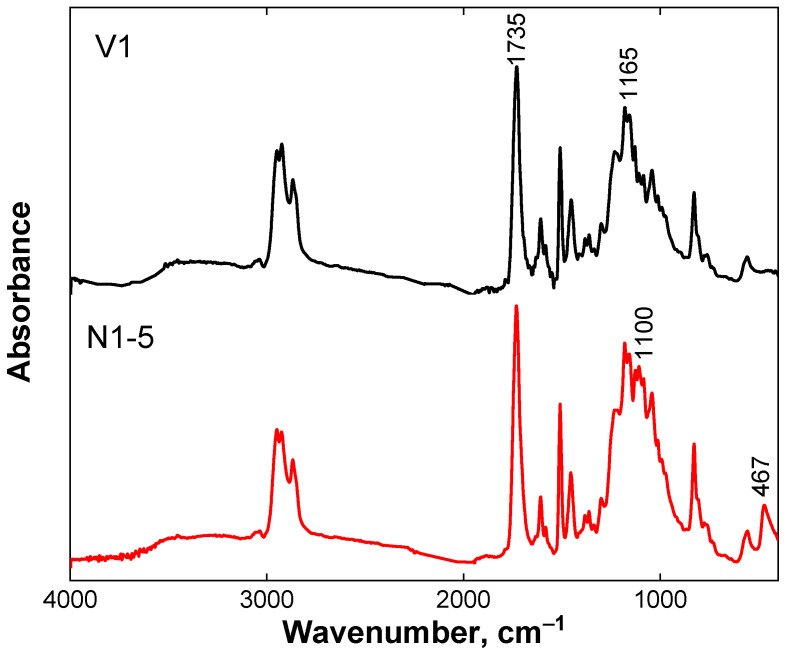
FTIR spectra for epoxy/silica vitrimer nanocomposite N1-5 with 5 wt% silica nanoparticles (red curve) and of the epoxy vitrimer V1 prepared under the same conditions, but without nanoparticles (black curve).

Figure 2 shows the FTIR spectra of the V1 and N1-5 samples. In the spectra, one can see the absorption bands of ester groups at 1735 and 1165 cm^−1^ (stretching vibrations of C=O and C–O–C bonds, respectively [36]) arising as a result of the reaction of DGEBA epoxy groups with anhydride MHHPA. At the same time, the characteristic band of the epoxy ring vibration of DGEBA at 916 cm^−1^ (stretching vibrations of C–H bonds of epoxy group) as well as the intense bands of the hardener MHHPA at 1780 and 1858 cm^−1^ (asymmetric *ν*_as_ and symmetric *ν*_s_ stretching vibrations of C=O bond of anhydride group [37]) and at 892 cm^−1^ (stretching vibrations of C–O bond of anhydride group [38]) are absent. These data evidence that the anhydride groups of MHHPA reacted with epoxy groups of DGEBA giving the ester bonds. The appearance of broad absorption between 1000 and 1200 cm^−1^, culminating at 1100 cm^−1^, and the signal located at 467 cm^−1^, corresponding to asymmetric bending vibrations of Si–O–Si bonds [39], proves the incorporation of silica nanoparticles into the epoxy vitrimer matrix.

**Figure 3:**
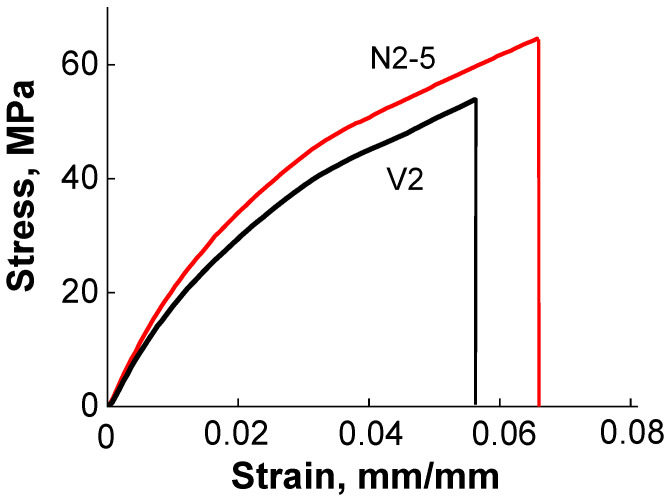
Tensile test for epoxy/silica vitrimer nanocomposite N2-5 with 5wt% silica nanoparticles (red line) and of the epoxy vitrimer V2 prepared under the same conditions, but without nanoparticles (black line).

The mechanical properties (elastic modulus, tensile strength, and elongation at break) of the epoxy/silica vitrimer nanocomposites were compared with the properties of the epoxy vitrimers prepared by thermal curing of DGEBA in the presence of MHHPA and ZAA under similar conditions but without silica nanoparticles. It was shown that the introduction of 5–10 wt% of silica nanoparticles in the vitrimer matrix increases the elastic modulus (Table 1, Figure 3). Similar behavior was observed for DGEBA/MHHPA/silica nanocomposites prepared in the absence of transesterification catalyst [24,40,41,42]. The increase of the modulus at the addition of silica nanoparticles was explained by much higher modulus of silica (*G* = 29.91 GPa [43], *E* = 70 GPa [41]) compared to the epoxy matrix and the restriction of polymer chain mobility by nanoparticles. The same reasons seem to be valid for epoxy/silica vitrimer nanocomposites under study.

The empirical Guth equation [44] was used to estimate the Young’s modulus *E*_c_ of epoxy/silica vitrimers at different content of silica nanoparticles:

(2) $$\frac{E_{c}}{E_{m}}=1+2.5V_{p}+14.1V_{p}^{2}$$

where *V*_p_ is the particle volume fraction, and *E*_c_ and *E*_m_ are the Young’s modulus of the composite and the epoxy matrix, respectively. The results are presented in Table 1. It is seen that the experimental *E*_c_ values slightly exceed the calculated ones. Similar behavior was observed for soft epoxy/silica vitrimers [18]. It was attributed to the aggregation of nanoparticles in the epoxy matrix [18].

The increase of the elastic modulus with increasing content of nanoparticles was also found for other vitrimer nanocomposites [19]. Note that to increase the strength of nanocomposites the nanoparticles should interact with the matrix so that the applied stress could be effectively transferred from the matrix to the particles [45]. In the present system, such interactions may be due either to the hydrogen bonding or to transesterification reaction between OH-groups of silica and ester groups of polymer leading to covalent attachment of particles to polymer matrix.

Table 1 and Figure 3 show that the addition of silica nanoparticles increases the tensile stress of vitrimer nanocomposites. Therefore, silica particles make the vitrimer stronger. For instance, in the second series of samples (V2 and N2-5) 5 wt% of silica induces the augmentation of the tensile stress from 54 to 65 MPa (Figure 3), i.e., by 20%. This is close to the results for DGEBA/MHHPA/silica nanocomposites prepared in the absence of transesterification catalyst, for which 5.3 wt% of silica induce the increase of tensile strength by ca. 16% [46]. The observed effect is expected [45] to be mainly due to polymer-particle interactions strengthening the network. At the same time, in soft epoxy vitrimersat room temperature the tensile stress decreases upon addition of silica from 25 to 11 MPa [18]. Therefore, in the present paper we first show that silica particles can strengthen hard epoxy vitrimers by increasing their tensile stress like in the case of ordinary epoxy thermosets, which do not undergo chain exchange reactions.

Figure 3 demonstrates that in the second series of samples (V2 and N2-5) the introduction of silica nanoparticles in the vitrimer matrix increases the elongation at break δ_B_ from 5.6 to 6.5%. Similar behavior of DGEBA/MHHPA/silica thermoset nanocomposites without transesterification catalyst [42], for which δ_B_ augments from 2.7 to 4.8% upon addition of 6 wt.% of silica, was attributed to lower crosslinking density of epoxy matrix [42] in the presence of nanoparticles hindering cure reaction. Most probably, the same reason is valid for the vitrimer nanocomposite N2-5 under study. By contrast, in the first series of samples (V1, N1-5 and N1-10, Table 1) synthesized at lower DGEBA concentration the elongation at break decreases from 7.3 to 4.9% in the presence of nanoparticles, similar to the case of soft epoxy/silica vitrimer nanocomposites, where also a two-fold decline of the elongation at break was observed [18]. Such behavior can be related to the attraction between silica particles and polymer matrix. These results suggest that in more dilute system, the nanoparticles do not significantly deteriorate the formation of the epoxy network, and the polymer-particle attraction becomes a dominant factor affecting the elongation at break.

**Figure 4:**
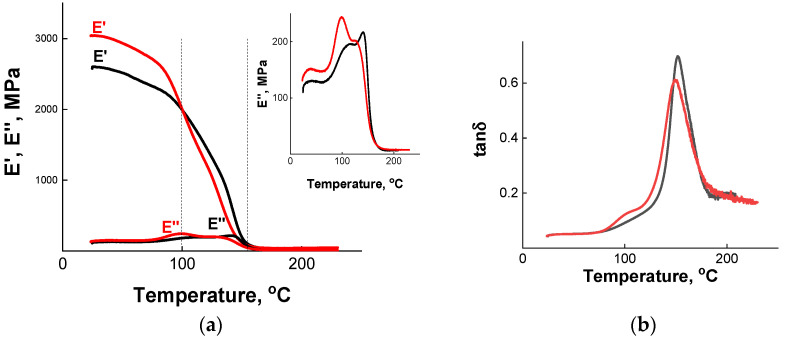
Temperature dependence of storage *E*′ and loss *E*″ moduli (**a**) and of loss tangent tan δ (**b**) for epoxy/silica vitrimer nanocomposite N2-5 with 5 wt% silica nanoparticles (red curve) and of the epoxy vitrimer V2 prepared under the same conditions, but without nanoparticles (black curve).

Dynamic mechanical tensile tests at the frequency of 1 Hz were performed. The resulting temperature dependences of storage *E*′ and loss *E*″ moduli and mechanical loss tangent tan δ are presented in Figure 4. A peak observed on tan δ curve (Figure 4b) determines the α-transition of the cured epoxy network [26]. It indicates that the glass transition temperatures of the nanocomposite and the neat matrix without nanoparticles (samples N2-5 and V2, Table 1) are equal to 150 and 152 °C, respectively. Note that the peak has a small shoulder at temperature around 100 °C, which is more pronounced in nanocomposite as compared to neat epoxy matrix. It may be caused by the fragments of the incompletely formed network as was shown theoretically [47]. Such behavior can result from hindering of the proper curing of polymer caused by nanoparticles. It is interesting that in soft epoxy/silica vitrimer nanocomposites [18] two peaks on the tan δ curves were also observed, but the peak at lower temperature was larger and it was attributed to the α-transition. The smaller peak was not discussed [18].

Figure 4a shows that *E*′ is much larger than *E*″ at all studied temperatures, which is expected. At 25 °C, the storage modulus *E*′ of the nanocomposite is equal to 3 GPa, which is appreciably higher than for the neat matrix (2.6 GPa). One can see (Figure 4a) that the storage modulus *E*′ decreases at heating both for nanocomposite and for the unfilled vitrimer, which can be due to the softening of the polymer matrix at increasing temperatures. In addition, in the case of nanocomposites, some contribution to the lowering of *E*′ comes from the difference between the CTE of the polymer matrix and the silica particles, which induces relaxations in the polymer phase, as was suggested previously [48,49]. Note that below 100 °C, the *E*′ of the nanocomposite is higher than that of the unfilled vitrimer matrix *E*′. When the temperature rises above 100 °C, the situation changes: the *E*′ of the nanocomposite becomes lower. For instance, at 130 °C the storage modulus equals to 0.82 and 1.16 GPa in the presence and in the absence of nanoparticles, respectively. Finally, in the temperature range from 160 to 250 °C, the difference between both samples vanishes. Therefore, the reinforcing effect of the filler is observed mainly in the glassy state. Probably, when the system approaches *T*_g_, enhanced mobility of polymer chains reduces the adhesion of polymer to the nanoparticles [50]. As a result, the contribution of polymer-particle interactions increasing *E*′ becomes lower than the contribution of polymer defects formed at the network synthesis in the presence of nanoparticles, which decreases *E*′. Similar behavior was found for epoxy/graphene oxide vitrimer nanocomposites [19]. In this case, below 60 °C the storage modulus *E*′ of nanocomposites was higher than for neat matrix by up to 30%, whereas above this temperature, the situation was reversed, and *E*′ of nanocomposite became up to 6-fold lower than for the neat matrix. Note that in the present system, the decrease of *E*′ of the nanocomposite with respect to neat matrix is much less pronounced.

Higher mobility of polymer chains in the vicinity of *T*_g_ induces the rise of the loss modulus *E*″ (Figure 4a). From Figure 4a (inset) one can see that the temperature dependence of *E*″ demonstrates two loss maxima: the first one (low-slopped, low-temperature) occurs at 96 and 107 °C, the second one (high-temperature) at 137 and 146 °C for nanocomposite and unfilled vitrimer, respectively. The first maximum at about 100 °C corresponding to the shoulder on the tan δ curve seems to be caused by the fragments of the incompletely formed network as was discussed above. The second (high-temperature) peak of losses associated with a sharp increase of tan δ (Figure 4b) is due to α-transition (glass transition) of the cured epoxy network [46,47]. Note that *T*_g_ for the nanocomposite is somewhat lower than for the unfilled network, which may also be due to effective preventing of the complete curing of the epoxy matrix by silica nanoparticles [47]. According to literature data [19], small lowering of *T*_g_ can be helpful to achieve a low temperature self-healing of the material. One can suggest that it may be also beneficial for low temperature welding.

**Figure 5:**
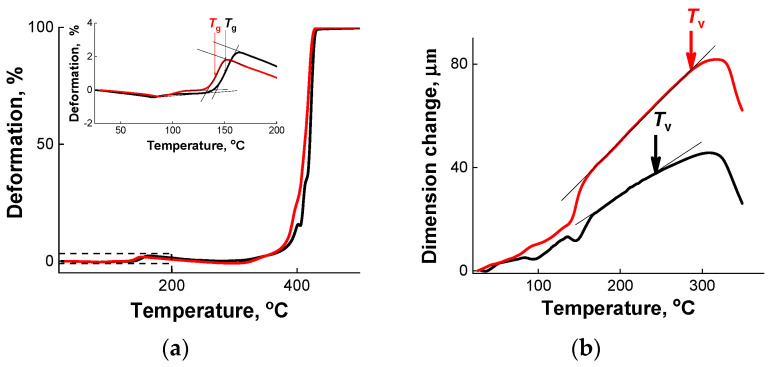
Thermomechanical curves (**a**) and expansion as a function of temperature (**b**) for epoxy/silica vitrimer nanocomposite with 5 wt% silica nanoparticles (sample N2-5) (red curve) and for the epoxy vitrimer prepared under the same conditions, but without nanoparticles (sample V2) (black curve).

TMA was used to compare the thermal properties (*T*_g_, *T*_v_, CTE, and degradation temperature *T*_d_) of the epoxy/silica vitrimer nanocomposites with those of the neat epoxy vitrimer. The data are shown in Figure 5 and Table 2. The first temperature transition (*T*_g_) is associated with devitrification of the material [51]. The second temperature transition (*T*_v_) appears only in the vitrimers [1,3] and reflects defrosting of the topology of polymer networks when the sample is heated. A third transition (*T*_d_) associated with the thermal degradation of the samples is observed at a high temperature [52].

The values of *T*_g_ were determined from the inflection point of the temperature transition of epoxy/silica thermosets from the glassy to the rubbery state (Figure 5a, inset). Figure 5a and Table 2 show that the addition of SiO_2_ nanoparticles slightly diminishes the *T*_g_ values, which is consistent with DMA data (Figure 4b). The higher the content of silica the more pronounced is the decrease of *T*_g_ (Table 2). Quite similar lowering of *T*_g_ values from 144 down to 132 °C upon increasing silica content from 0 to 5.3 wt.% was found previously for DGEBA/MHHPA/silica nanocomposites prepared in the absence of transesterification catalyst [46]. Similar behavior was also observed for epoxy/graphene oxide vitrimer nanocomposites [18]. As stated above, the decrease in *T*_g_ may be due to the formation of loose structures [15] when the filler creates steric hindrances for the synthesis of the epoxy network. This contrasts with the behavior observed in soft epoxy/silica nanocomposites, where the *T*_g_ slightly increases upon addition of nanoparticles [18]. It indicates that nanoparticles do not interfere the network formation when it is loose. However, when the network is dense (like in the hard thermosets under study), the nanoparticles may hinder the proper cross-linking. This is consistent with the conclusion made above from the analysis of the elongation at break data for soft and hard vitrimer nanocomposites.

The values of *T*_v_ temperature were determined according to ref. [1] from a sharp increase in CTE upon heating the sample (Figure 5b), which arises when the exchange reactions make the network more expandable. Our experiments were performed in the penetration mode. In this mode, when the sample is not very soft, its positive deformation is followed by the displacement of the punch, but when the sample becomes very soft (above *T*_v_), the sample expands and the course of deformation during punch penetration becomes negative (Figure 5b). The results of *T*_v_ determination are presented in Table 2. It is seen that all samples under study (both in the presence and in the absence of nanoparticles) demonstrate a *T*_v_ transition inherent to vitrimers [1,3]. It indicates that the addition of silica does not prevent the transesterication reaction, and the composite should be malleable at high temperature. Note that *T*_v_ slightly increases upon incorporation of silica particles. It means that the nanoparticles may stabilize the network topology, and the system needs to be heated more to unfreeze its topological structure. The found *T*_v_ values for vitrimers (*T*_v_ = 230 and 244 °C for V1 and V2) slightly exceed *T*_v_ = 210 °C for a sample obtained by curing DGEBA in the presence of glutaric anhydride and ZAA and determined by dilatometry at heating rate of 5 K/min [3]. The observed small difference in the values of *T*_v_ can be due to larger amount of catalyst in the sample reported in [3] (5 mol%), which makes the exchange reactions faster.

A sharp rise in deformation to 100% (Figure 4a) when the sample is heated at high temperatures (in the region about 400 °C) indicates the temperature of the onset of intensive thermal degradation *T*_d_. Table 2 shows that the *T*_d_ temperature practically does not change at the introduction of SiO_2_ nanoparticles. This is consistent with the literature data [15] for epoxy/carbon fibers nanocomposites.

From Table 2 one can see that the CTE values in the rubbery state (above *T*_g_) are higher than those below *T*_g_ since the rubbery material possesses more free volume allowing higher expansion. When the number of added nanoparticles is rather high (10%), they reduce the CTE values especially in the rubbery state (above *T*_g_). Similar behavior was observed for other epoxy/silica nanocomposites [24,43,53]. It was attributed to much lower CTE of the silica nanoparticles as compared to the neat epoxy resin. Additionally, one can expect that interactions of the particles with the epoxy network can restrict the mobility and deformation of the matrix thereby preventing the expansion of the resin matrix upon heating.

Thus, the effect of nanoparticles on the mechanical behavior of epoxy vitrimers at room temperature is quite similar to that observed in the corresponding epoxy thermosets without transesterification catalyst. It concerns, in particular, the increase of the elastic modulus and the tensile strength and the decrease of CTE induced by added nanoparticles. It indicates that nanoparticles make the polymer material stronger and enhance its dimensional stability at heating.

### 3.2. Investigation of the Ability of Epoxy/Silica Vitrimers for Welding

**Figure 6:**
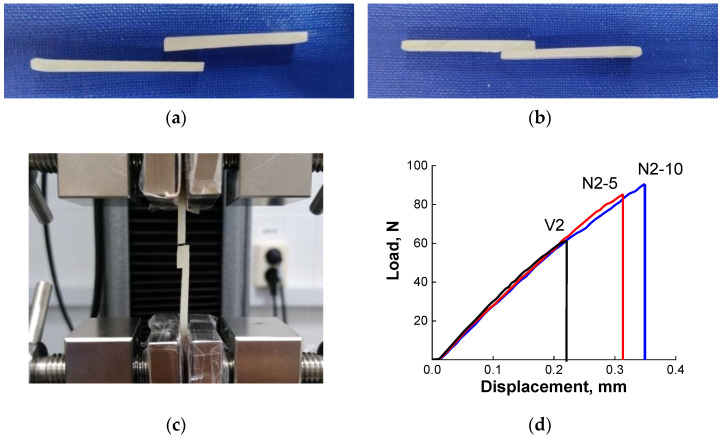
Photographs of the silica/epoxy vitrimer nanocomposite (sample N2-5) before (**a**) and after (**b**) welding at 160 °C for 6 h and after lap-shear test of the welded sample (**c**). Stress–strain curves (**d**) of epoxy/silica vitrimer samples at different content of nanoparticles: 0 (sample V2), 5 (sample N2-5) and 10 wt% (sample N2-10) welded under similar conditions (160 °C, 5 h).

The welding of epoxy/silica vitrimers was performed by a compress molding. In these experiments, two vitrimer samples (1.8 × 4 × 37 mm^3^) were joined together with an overlap area of 25 mm^2^ for few hours under pressure (Figure 6a,b). A welding pressure is necessary because it increases the real contact area on the interface, which is especially important, when the interface is rough. The welding was performed at 160 °C, that is above *T*_g_, but below topology freezing temperature *T*_v_. In the vitrimer, there are some chain exchange reactions even below *T*_v_ and we would like to check, whether they can be sufficient to provide a welding in the present system. Taking into account that the temperature of the welding was well below *T*_v_ the time of welding was rather long (5–6 h).

It was shown that this time was enough to weld all the vitrimer samples under study. The adhesion increases with increasing the welding time. For example, samples V2 and N2-5 welded during 5 h disrupt at the welded joint in contrast to the same samples welded during 6 h, which break outside the overlapped part (Table 3). Therefore, the welding effect can be effectively improved by properly increasing the welding time. In further experiments, the welding time was fixed at 6 h.

The effect of silica nanoparticles on the ability of epoxy vitrimers for welding and on the strength of the welded joints was studied by tensile test experiments. The results are summarized in Table 3. It is seen that the strength of the welded joint depends on the concentration of silica nanoparticles. For instance, for a first series of samples (V1, N1-5 and N1-10) the welded vitrimer without nanoparticles disrupts in the welding joint at application of 78 N, whereas in the corresponding nanocomposites the rupture occurs outside the welded joint (Table 3). This result indicates to a higher strength of the polymer matrix at the welding site, when nanoparticles are added. Therefore, silica nanoparticles considerably increase the strength of a joint. The positive effect of nanofiller (graphene oxide) on the self-healing properties and shape memory of epoxy vitrimers was discovered by Krishnakumar et al. [19]. Here, we demonstrate similar effect of filler on welding ability. Surprisingly, an increase of the concentration of silica from 5 to 10 wt% diminishes the strength of the joint from 119 to 46 N. It may be due to the presence of some bubbles in this particular sample.

In the second series of samples (V2 and N2-5) prepared at higher monomer concentration both the neat vitrimer and the nanocomposite form rather strong welded joint, which withstands the load (Figure 6c). However, the nanocomposite vitrimer is always stronger (Table 3): it breaks at almost 2-fold higher load than the corresponding neat vitrimer. It means that nanoparticles strengthen the welding joint. Similar conclusion can be drawn from the tensile tests of welded specimens of the third series cured for shorter time (5 h) (Table 3, Figure 6d). Although they are broken at the joint, the breaking load increases with increasing content of nanoparticles. Therefore, the results obtained unambiguously show that nanoparticles significantly increase the strength of the welding joint.

**Figure 7:**
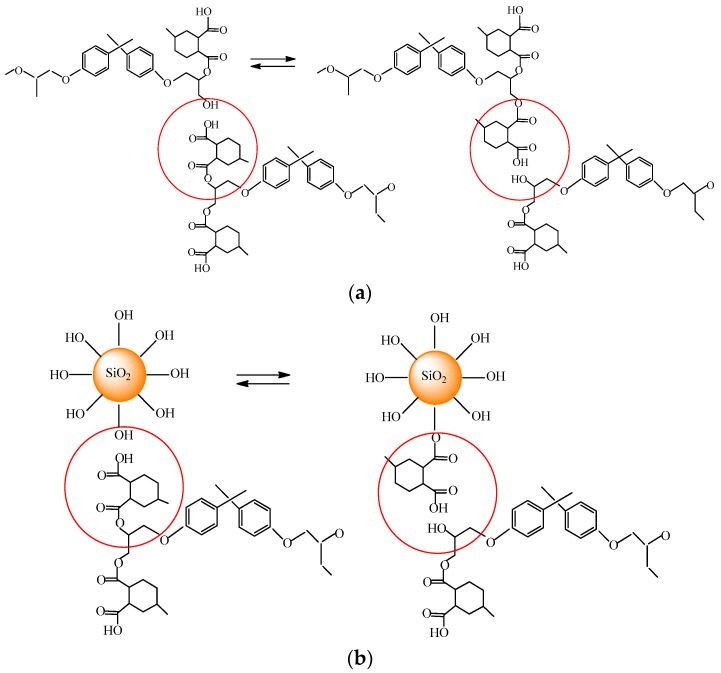
Schematic representation of transesterification reaction between epoxy chains (**a**) and on the epoxy/silica interface (**b**).

The observed behavior can be explained as follows. According to multiscale modeling of surface welding of thermally induced dynamic covalent network polymers [12], at the beginning of surface welding, an initial chain density gradient drives the polymer chains to diffuse to the interface, where there is a lack of polymer. Then, at the interface the bond exchange reactions between polymer chains belonging to different surfaces occur thereby bridging the two samples together. In the present system, the interchain exchange is the transesterification, which proceeds between ester and hydroxyl groups (Figure 7) producing a new ester and a new hydroxyl group. Increase of the strength of the joint upon addition of nanoparticles indicates that silica particles participate in the welding. They can contribute to the attraction between polymer chains belonging to different welding surfaces by forming hydrogen bonds with them. Moreover, one can suggest that the transesterification reactions can occur not only between epoxy chains, but also on the epoxy/silica interface.

Note that in the present study, the welding temperature is well below the *T*_v_. Nevertheless, welding occurs, indicating that some chain exchange reactions proceed even at these conditions, but these reactions are not numerous and therefore the welding requires rather long time. The occurrence of the chain exchange reactions even below *T*_v_ can be supported by the fact that epoxy/silica nanocomposite and the corresponding epoxy thermoset (without interchain exchange catalyst) do not weld at 160 °C at all. Therefore, the presence of interchain exchange catalyst is ultimate to weld the samples. The welding of thermoset vitrimer below *T*_v_ was previously demonstrated on an example of DGEBA cured with glutaric anhydride in the presence of ZAA [1]. The *T*_v_ for this vitrimer was approximately 210 °C [3], and the welding of two samples was performed at 150 °C [1]. In the present paper, for the first time we show that silica nanoparticles promote the welding of vitrimers and make the welding joint stronger.

## 4. Conclusions

For the first time, hard epoxy/silica thermoset nanocomposites that can change their topology when heated in the presence of interchain exchange catalyst ZAA have been prepared. The topology of the epoxy network is changed as a result of the transesterification reaction via the interaction of carbonyl groups –C=O of ester with hydroxyl groups of products of incomplete curing of the epoxy monomer.

It is shown that in the new nanocomposites, nanoparticles strengthen the vitrimers by increasing their tensile stress from 48 to 60 MPa and elastic modulus from 1.8 to 2.6 GPaand simultaneously improve their dimensional stability at heating, since the CTE in the presence of nanoparticles decreases. This is the first demonstration of the fact that silica particles can reinforce hard epoxy vitrimers by increasing their tensile stress.

By dynamic mechanical analysis it was shown that the reinforcing effect of particles is mainly observed in the glassy state. When the system approaches *T*_g_, enhanced mobility of polymer chains reduces the adhesion of polymer to the nanoparticles thereby diminishing the contribution of polymer-particle interactions to the mechanical properties.

By thermomechanical analysis it was shown that all samples under study (both in the presence and in the absence of nanoparticles) demonstrate a *T*_v_ transition inherent to vitrimers indicating that silica particles do not prevent the transesterification reaction. At the same time, nanoparticles slightly increase the topology freezing temperature *T*_v_ suggesting that they may stabilize the network topology.

Both by dynamic mechanical and thermomechanical analyses it was evidenced that the addition of SiO_2_ slightly diminishes the glass transition temperature of the vitrimers. It was attributed to the formation of loose structures when the incorporated particles hinder the cure reaction. Small decrease of *T*_g_ can be beneficial for low temperature welding.

One of the most important findings of this study is the observation that the silica particles enhance the welding of vitrimer samples induced by interchain exchange reactions and make the welding joints stronger. To the best of our knowledge, the last effect of silica particles on the welding was first observed in vitrimers. It opens up wide perspectives for assembling and joining thermoset composites to produce novel repeatedly recyclable vitrimer materials with a combination of desirable properties provided by each of the welded components.

## Figures and Tables

**Table 1:** Conditions of preparation and mechanical properties of epoxy/silica vitrimer nanocomposites.

Sample	[DGEBA]/[MHHPA]/[ZAA], mol/mol/mol	[SiO_2_], wt%	Tensile Stress, MPa	Tensile Strain, %	Elastic Modulus, GPa	
					Tensile Tests	Guth’s Prediction
V1	0.5/1.0/0.05	0	48	7.3	1.8	
N1-5	0.5/1.0/0.05	5	57	5.6	2.4	2.0
N1-10	0.5/1.0/0.05	10	60	4.9	2.6	2.2
V2	0.72/1.0/0.043	0	54	5.6	2.0	
N2-5	0.76/1.0/0.043	5	65	6.5	2.3	2.2
N2-10	0.76/1.0/0.04	10	68	6.5	2.4	2.3

**Table 2:** Thermal properties of epoxy/silica vitrimer nanocomposites determined from TMA.

Sample	Glass Transition Temperature *T*_g_, °C	Topology Freezing Temperature *T*_v_, °C	Coefficient of Thermal Expansion CTE × 10^6^, μm/(m K)		Temperature of Onset of Thermal Degradation *T*_d_, °C
			Before *T*_g_	Above *T*_g_	
V1	141	230	70	230	396
N1-5	136	280	70	205	396
N1-10	130	290	64	150	395
V2	152	244	70	210	414
N2-5	144	289	70	200	405

**Table 3:** Tensile tests of welded specimens.

Sample	[SiO_2_], wt%	Welding Time, h	Load, N	Rupture Site
V1	0	6	78	Welding joint
N1-5	5	6	119	Beyond the weld
N1-10	10	6	46	Beyond the weld
V2	0	6	44	Beyond the weld
N2-5	5	6	71	Beyond the weld
V2	0	5	61	Welding joint
N2-5	5	5	85	Welding joint
N2-10	10	5	91	Welding joint

## Data Availability

The data presented in this study is openly available.

## Supplementary Materials

The following are available online at https://www.mdpi.com/article/10.3390/polym13183040/s1, Figure S1: Schematic representation of mechanism of noncatalyzed reaction of aromatic epoxides such as diglycidyl ether of bisphenol A.
