# Supplementary material for: Unmodified Silica Nanoparticles Enhance Mechanical Properties and Welding Ability of Epoxy Thermosets with Tunable Vitrimer Matrix
Source: Polymers (Basel). 2021 Sep 9;13(18):3040. doi: 10.3390/polym13183040 (PMC8467415; doi:10.3390/polym13183040)
Supplement: Supplementary file 1 [file polymers-13-03040-s001.zip › polymers-1342367-supplementary.pdf]

polymers

Supplementary Materials

Unmodified Silica Nanoparticles Enhance Mechanical Properties and Welding Ability of Epoxy Thermosets with Tunable Vitrimer Matrix

Anna I. Barabanova [1,*], Egor S. Afanas`ev [1], Vyacheslav S. Molchanov [2], Andrey A. Askadskii [1,3] and Olga E. Philippova [2]

1 A.N. Nesmeyanov Institute of Organoelement Compounds, Russian Academy of Sciences, 119991 Moscow, Russia; barabanoval@polly.phys.msu.ru (A.I.B.); nambrot@yandex.ru (E.S.A.); andrey@ineos.ac.ru
2 Physics Department, Moscow State University Moscow State University, 119991 Moscow, Russia; molchan@polly.phys.msu.ru (V.S.M.); phil@polly.phys.msu.ru (O.P.)
3 Moscow State University of Civil Engineering, 129337, Russia
* Correspondence: barabanova@polly.phys.msu.ru

Citation: Barabanova, A.I.; Afanas, E.S.; Molchanov, V.S.; Askadskii, A.A.; Philippova, O.E. Unmodified Silica Nanoparticles Enhance Mechanical Properties and Welding Ability of Epoxy Thermosets with Tunable Vitrimer Matrix. *Polymers* **2021**, *13*, 3040. https://doi.org/10.3390/polym13183040

Received: 30 July 2021
Accepted: 30 August 2021
Published: 9 September 2021

Publisher's Note: MDPI stays neutral with regard to jurisdictional claims in published maps and institutional affiliations.

Copyright: © 2021 by the authors. Licensee MDPI, Basel, Switzerland. This article is an open access article distributed under the terms and conditions of the Creative Commons Attribution (CC BY) license (https://creativecommons.org/licenses/by/4.0/).

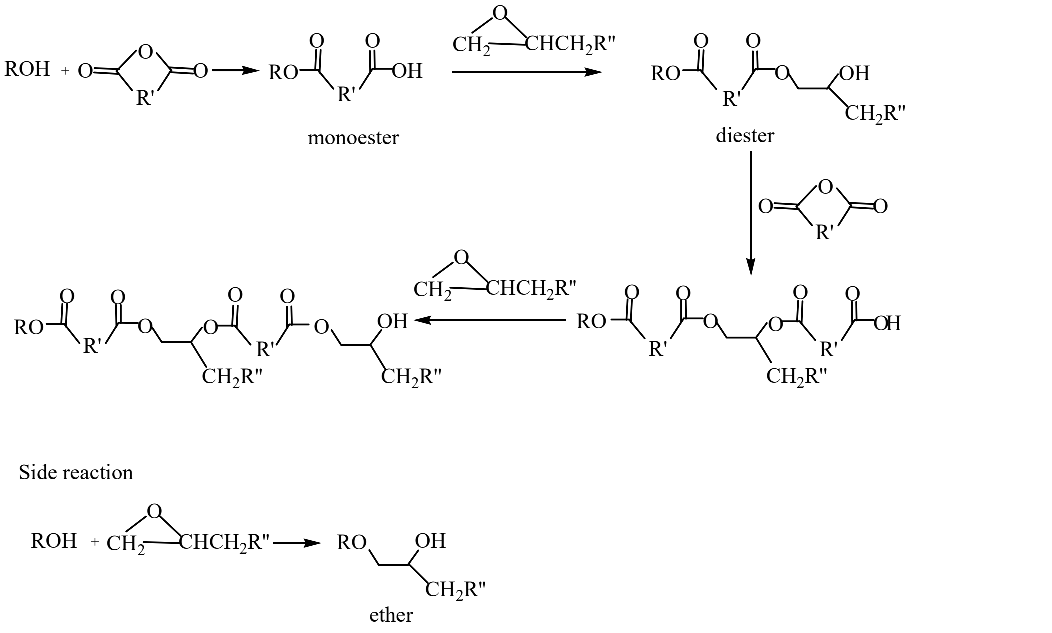

Figure S1. Schematic representation of mechanism of noncatalyzed reaction of aromatic epoxides such as diglycidyl ether of bisphenol A.
